# Genetically Confirmed Wilson Disease: A Retrospective Cohort Study From Bahrain

**DOI:** 10.7759/cureus.71805

**Published:** 2024-10-18

**Authors:** Hasan M Isa, Fawzeya A Alahmed, Maryam Y Busehail, Zahra H Isa, Kawthar M Abdulla

**Affiliations:** 1 Department of Pediatrics, Arabian Gulf University, Manama, BHR; 2 Department of Pediatrics, Salmaniya Medical Complex, Manama, BHR

**Keywords:** bahrain, ceruloplasmin, chelating agents, copper, genetic mutations, hepatic dysfunction, hepatolenticular degeneration, penicillamine, wilson disease

## Abstract

Introduction

Wilson disease (WD) is a rare inherited autosomal recessive disorder caused by a mutation in the *ATP7B* gene. This mutation affects copper metabolism, leading to the accumulation of copper in the liver, brain, cornea, and other tissues. If not treated, WD can lead to significant morbidities. This study aimed to display the prevalence, clinical presentations, diagnosis, treatment, and outcome of WD in Bahrain.

Methods

This is a retrospective cohort study of patients diagnosed with WD in the Department of Pediatrics, Salmaniya Medical Complex, Manama, Kingdom of Bahrain, from March 2002 to July 2024. The diagnosis of WD was based on clinical presentations, laboratory markers, radiological imaging, and genetic testing.

Results

Up to July 2024, eight patients from four families were diagnosed with WD in Bahrain. Accordingly, the prevalence of WD was 1.7 patients per 100,000 (0.002%). Parental consanguinity was noted in all families. Males were equal to females (n=4, 50% each). The mean age at presentation was 13 ± 3.6 years, ranging from 9 to 21 years. Three patients (37.5%) presented with hepatic manifestations, two (25%) with neurological manifestations, and three (37.5%) were detected upon family screening. Kayser Fleischer (KF) rings were found in two (25%) patients via slit lamp examination. Of the seven patients with available data, five (71.4%) had low serum copper, six (85.7%) had low serum ceruloplasmin, and six (85.7%) had high 24-hour urinary copper. Genetic testing was positive for the *ATP7B* gene variants in all patients, of which four members of one family had a novel mutation. Abdominal ultrasound revealed hepatic cirrhosis in four (50%) patients, increased hepatic echogenicity in five (62.5%), splenomegaly in three (37.5%) patients, and ascites in one (12.5%) patient. Brain magnetic resonance imaging (MRI) was positive in three (50%) out of six patients. All patients received copper chelating therapy. Six (75%) patients survived; one of them underwent living-related liver transplantation due to rapidly progressive neurological symptoms. Two (25%) patients died due to hepatic failure and encephalopathy.

Conclusion

WD is a rare disorder in Bahrain. This study presents eight patients with WD from four families. Five patients were symptomatic at presentation, while three were diagnosed upon genetic family screening. Hepatic manifestations were the most common presentation, followed by behavioral changes and neurological symptoms. Two patients had KF rings, while three had classical brain MRI findings. Genetic testing served as a confirmatory diagnostic tool that revealed a diversity of variants causing the disease, of which one variant was a novel mutation. Despite oral chelating therapy, morbidity and mortality remain high in patients with WD.

## Introduction

WD is a rare inherited autosomal recessive disorder, also known as hepatolenticular degeneration [1]. The prevalence of WD was estimated to be between 1/40,000 and 1/100,000 [1]. Yet, this prevalence varies by region.

Different types of variants and genetic heterogeneity are involved in causing WD [1,2]. Pathogenic variants in the P-type ATPase copper transporting gene ATP7B result in the development of the disease [3,4]. These variants affect copper metabolism by disrupting copper transport, resulting in excessive copper deposition in different body parts, particularly the liver, brain, and cornea [3]. Until 2023, 1275 distinct ATP7B gene variants were analyzed and identified by molecular genetics [5].

WD has a variety of clinical presentations that can be affected by race and region [1,2]. Therefore, WD can present with hepatic, neurological, hematological, and renal symptoms and signs [3]. Hepatic and hematological manifestations usually appear at the beginning of the disease, while neurological symptoms might come later [1].

Diagnosis of WD is based on clinical findings, physical examination, and laboratory investigations [1,3]. Thus, abnormalities in specific biochemical markers such as low serum ceruloplasmin level, high urinary copper, and increased hepatic copper concentration can be used to establish the diagnosis [6,7]. However, the detection of ATP7B gene variants via genetic testing is the gold standard [8-10].

It is important to recognize the signs and symptoms of WD early to help in earlier treatment and a better prognosis of the disease [3]. The goal of WD treatment is to reach normal copper levels and preserve them [11]. This might be achieved via dietary restrictions on copper-containing foods along with copper-chelating agents [8,12]. WD can lead to significant morbidities if not treated [12].

WD has been reported in many countries worldwide [13]. However, this disease was not previously reported in Bahrain. Hence, this study aimed to display the prevalence, clinical presentations, diagnosis, treatment, and outcome of WD in Bahrain.

## Materials and methods

Study design, setting, and population

In this retrospective cohort study, all patients with WD who were diagnosed at the Department of Pediatrics, Salmaniya Medical Complex, Government Hospitals, Manama, Bahrain, from 1st March 2002 to 20th July 2024 were included. Electronic and paper-based medical records were reviewed. Salmaniya Medical Complex is the main tertiary hospital in Bahrain, and any patients with suspected WD are referred to it for diagnosis and management. 

The diagnosis of WD was based on a combination of clinical presentations, physical findings, laboratory investigations, genetic testing, and radiological imaging [6]. The diagnosis was in accordance with the latest three guidelines: the American Association for the Study of Liver Diseases (AASLD) guideline [8], the European Association for the Study of the Liver (EASL) guideline [9], and the European Society for Paediatric Gastroenterology, Hepatology, and Nutrition (ESPGHAN) guideline [10]. These three guidelines agreed that ATP7B variant detection by genetic testing is the gold standard for WD diagnosis [8-10]. In addition, they emphasized the importance of measuring 24-hour urinary copper excretion and quantification of liver copper via liver biopsy or noninvasive methods, while measuring serum ceruloplasmin level alone is not sufficient for the diagnosis as it can be normal in some patients [8-10].

Data collection

Patients’ demographic data, including sex, nationality, age of presentation, family history of WD, and clinical presentations, were collected. Laboratory findings including complete blood counts, liver function tests (serum total protein, albumin, globulin, total bilirubin, direct bilirubin, indirect bilirubin, alkaline phosphatase [ALP], aspartate aminotransferase [AST], alanine aminotransferase [ALT], and gamma-glutamyl transferase [GGT]), coagulation profiles, serum ceruloplasmin level, serum copper level, 24-hour urinary copper, and genetic testing were gathered. 

Genetic testing was conducted by one of two methods. The first method was the whole exome sequencing test, which was performed through the Centogene laboratory in Germany. The generated library was sequenced on an Illumina platform to obtain at least 20x coverage depth for > 98% of the targeted bases. The second method was the molecular genetic analysis of the ATP7B gene, which was performed through the Bioscientia laboratory in Germany. A multiplex polymerase chain reaction of the coding regions of the ATP7B gene (chromosome 13) and corresponding intron/exon boundaries (±20 bp) was conducted. Library preparation was performed by tagmentation (QXT, Agilent Technologies [California, USA]), followed by next-generation sequencing (MiSeq, Illumina [San Diego, USA]).

Radiological imaging results, including abdominal ultrasound (US), abdominal computed tomography (CT) scan, brain CT scan, and MRI, were noted along with liver biopsy histology and endoscopic findings. Medical and surgical therapies were reviewed. The follow-up period and the patients' outcomes were assessed. The patients' outcomes included survival, development of complications, need for liver transplantation, and mortality.

Statistical analysis

The patients' data were initially entered into a Microsoft Excel 2016 sheet, then transferred to IBM Corp. Released 2012. IBM SPSS Statistics for Windows, Version 21.0. Armonk, NY: IBM Corp. for analysis. The frequencies and percentages were calculated for categorical variables. Continuous variables were presented as mean ± standard deviation for normally distributed variables or median and interquartile range for non-normally distributed variables.

Ethical clearance

This study was conducted in accordance with the principles of the Helsinki Declaration and was ethically approved by the Research and Research Ethics Committee, Government Hospitals, Manama, Kingdom of Bahrain (IRB number: 11140223, 14th February 2023).

## Results

**Figure 1:**
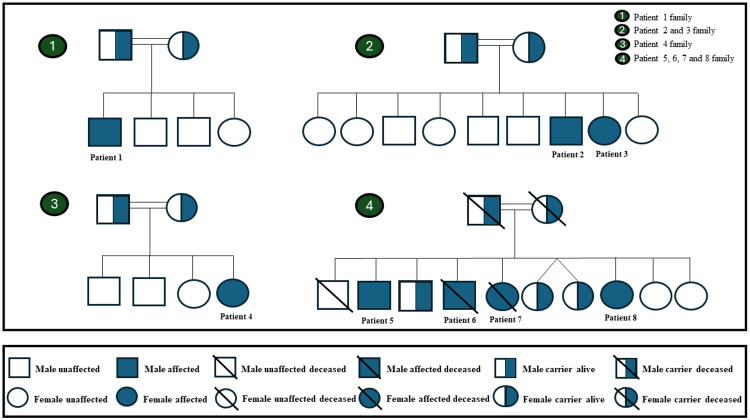
Family pedigrees of patients with Wilson disease. Image credits: Hasan M. Isa and Fawzeya A. Alahmed

Until July 2024, a total of eight patients from four families were diagnosed with WD. Family 1 had one patient (Patient 1), Family 2 had two (Patients 2 and 3), Family 3 had one patient (Patient 4), and Family 4 had four patients (Patients 5, 6, 7, and 8). Parental consanguinity was noted in all families (Figure 1).

Based on Bahrain's health statistics, the total population of Bahrain in 2020 was 1,472,204, and the population at risk (up to the age of 19 years) was 481,819 [14]. Accordingly, the prevalence of WD in Bahrain is 1.7 patients per 100,000 (0.002%).

As for family history, Patient 1's grandmother has a history of hepatitis C infection, but the patient tested negative. In Family 2, Patient 2's siblings were laboratory and genetically screened for the disease. As a result, his younger sister was found to have high liver enzymes, and WD was confirmed genetically (Patient 3). Their father has hypertension and diabetes mellitus, while their mother has hypercholesterolemia. The history of Family 3 was unremarkable. In Family 4 (Patients 5, 6, 7, and 8), three members died: the father had colon cancer, the mother had gastric cancer, and the elder brother died in a car accident.

Demographic data, clinical presentations, and physical findings are shown in Table 1.

**Table 1:** Clinical features of patients with Wilson disease. Data are presented as number (%), mean ± standard deviation, or median (interquartile range). WD: Wilson disease. KF: Kayser-Fleischer.

Clinical features	Patient 1	Patient 2	Patient 3	Patient 4	Patient 5	Patient 6	Patient 7	Patient 8	Total results
Sex (Male)	Male	Male	Female	Female	Male	Male	Female	Female	4 (50)
Country of origin (Bahraini)	Syria	Bahrain	Bahrain	Pakistan	Jordan	Jordan	Jordan	Jordan	2 (25)
Age of presentation (yr)	13	13	12	12	21	14	10	9	13 ± 3.6
Age at follow up (yr)	20	22	21	16	37	17 (died)	13 (died)	21	20.5 (16.3 - 21.8)
Family history of WD	No	Yes	Yes	No	Yes	Yes	Yes	Yes	6 (75)
The main clinical presentations	Hepatic symptoms	Neurological symptoms	Family screening	Hepatic symptoms	Family screening	Hepatic symptoms	Neurological symptoms	Family screening	5 (62.5)
Hepatic	Yes	Yes	No	Yes	No	Yes	Yes	Yes	6 (75)
Anorexia/reduced appetite	Yes	Yes	No	Yes	No	Yes	No	No	4 (50)
Abdominal pain	Yes	No	No	No	No	No	No	Yes	2 (25)
Jaundice	No	Yes	No	Yes	No	Yes	Yes	No	4 (50)
Hepatomegaly	Yes	Yes	No	Yes	No	Yes	No	No	4 (50)
Splenomegaly	No	No	No	No	No	No	No	No	0 (0.0)
Ascites	No	No	No	No	No	Yes	No	No	1 (12.5)
Neurological	No	Yes	Yes	No	No	Yes	Yes	No	4 (50)
Gait abnormality	No	Yes	No	No	No	Yes	Yes	No	3 (37.5)
Ataxia	No	Yes	No	No	No	Yes	Yes	No	3 (37.5)
Tremors	No	No	Yes	No	No	Yes	No	Yes	3 (37.5)
Speech changes	No	Yes	No	No	No	Yes	No	No	2 (25)
Tone	Normal	Hypotonia	Normal	Normal	Normal	Hypotonia	Normal	Normal	2 (25)
Power	Normal	Weak grip	Normal	Normal	Normal	Weak	Normal	Normal	2 (25)
Reflexes	Normal	Brisk	Normal	Normal	Normal	Brisk	Normal	Normal	2 (25)
Sensory loss	No	Yes	No	No	No	Yes	No	No	2 (25)
Psychiatric	No	Yes	Yes	No	No	Yes	Yes	No	4 (50)
Ophthalmic (KF ring)	No	Yes	No	No	No	Yes	No	No	2 (25)
Others									
School performance deterioration	No	Yes	Yes	No	No	Yes	Yes	Yes	5 (62.5)
Fever	Yes	No	No	Yes	No	No	No	No	2 (25)

Males were equal to females (n=4, 50% each). All the patients hold Bahraini citizenship; however, two (25%) are original Bahraini nationals, while six (75%) came from originally non-Bahraini families (four from a Jordanian family and one each from Syrian and Pakistani families). All the patients from the three non-Bahraini families were delivered in Bahrain, and their parents have lived in Bahrain for an extended period. Furthermore, the patients were diagnosed with WD, treated, and followed up in Bahrain. The mean age at presentation was 13 ± 3.6 years, ranging from 9 to 21 years.

At presentation, five (62.5%) patients were symptomatic, while three (37.5%) were diagnosed upon family screening. Three (37.5%) patients presented with hepatic symptoms, and two (25%) presented with neurological symptoms. 

Patient 1 presented with a low-grade fever, nausea, reduced appetite, and abdominal pain. His physical examination revealed spider nevi on his cheeks, abdominal distention, and hepatomegaly. Patient 2 was initially referred to psychiatric care as he presented with behavioral changes. He became notably introverted, and shy, and had a lack of concentration, along with significant weight loss over a duration of six months. Later on, he developed an abnormal gait. On examination, he had bilateral gynecomastia, hepatomegaly, wide-based gait, positive Romberg’s sign, signs of incoordination, slow slurred speech with dysarthria and stuttering, ataxia, hypotonia (right > left), weak hand grip (right > left), brisk reflexes, and diminished sensation of both feet. A slit lamp eye examination revealed bilateral KF rings. Patient 3 was diagnosed via screening after her sibling was diagnosed with WD (Patient 2). Initially, she was asymptomatic but failed her school exams and had a decline in memory and concentration. Later, she started to have tremors in her hands. She was not icteric and had no organomegaly. Patient 4 presented with fever, cough, epistaxis, reduced appetite, yellowish discoloration of skin and sclera, dark urine, itching, and urticarial rash a few days after coming back from Pakistan. She was jaundiced with hepatomegaly 2 cm below the costal margin, but her neurological examination was unremarkable. Patient 5 was asymptomatic and was diagnosed as part of family screening, as his three younger siblings were diagnosed before with WD (Patients 6, 7, and 8). His examination was unremarkable. He was diagnosed with type 2 diabetes mellitus and dyslipidemia at the age of 21 years. Patient 6 presented with yellowish discoloration of the skin and sclera. On examination, he had jaundice and hepatomegaly, along with bilateral KF rings. Patient 7 presented with an abnormal gait in addition to a deterioration of school performance. On examination, she had jaundice, incoordination of movements, and ataxia. Patient 8 was found to have abdominal pain along with deterioration of school performance at the time of family screening for the disease. She had an unremarkable physical examination.

Laboratory testing results were available in six patients but were missing in two (Patients 6 and 7). Patient 6 had cholestatic jaundice, elevated liver enzymes, low serum ceruloplasmin, high urine copper, and a negative hepatitis profile noted in his medical records, but the exact figures were not available (Table 2).

**Table 2:** Laboratory values of patients with Wilson disease. Data are presented as mean ± standard deviation, median (interquartile range), or number (%). WBC: white blood cells; ALP: alkaline phosphatase; AST: aspartate aminotransferase; ALT: alanine aminotransferase; GGT: gamma-glutamyl transferase; INR: International normalized ratio; APTT: activated partial thromboplastin time; AFP: alpha fetoprotein.

Laboratory marker	Normal range	Patient 1	Patient 2	Patient 3	Patient 4	Patient 5	Patient 6	Patient 7	Patient 8	Total results
WBC (x10^^9^/L)	3.6-9.6	11.9	2.9	6.03	14.02	7.01	NR	NR	7.08	8.2 ± 4.1
Hemoglobin (g/dL)	12-14.5	8.8	11.8	13	10.7	15.2	NR	NR	15.5	12.5 ± 2.6
Reticulocyte (%)	0.2-2.0	2.5	0.08	NR	12.4	NR	NR	NR	2.1	4.3 ± 5.5
Platelets count (x10^^9^/L)	150-400	375	48	180	199	272	NR	NR	344	236.3 ± 120.1
Total protein (g/L)	64-82	65	68	60	69	86	NR	NR	74	70.3 ± 8.9
Serum albumin (g/L)	38-50	23	36	45	26	50	NR	NR	45	37.5 ± 11.1
Serum globulin (g/L)	15-30	42	32	15	43	36	NR	NR	29	32.8 ± 10.3
ALP (U/L)	150-420	306	673	476	141	87	NR	NR	291	329.0 ± 217.2
AST (U/L)	10-40	NR	32	NR	NR	NR	NR	NR	61	46.5 ± 20.5
ALT (U/L)	<41	118	54	321	47	134	NR	NR	124	121.0 (52.3 - 180.8)
GGT (U/L)	<18	400	125	215	345	213	NR	NR	97	232.5 ± 119.4
Total bilirubin level (µmol/L)	<21	48	28	3	100	7	High	NR	5.4	31.9 ± 37.6
Direct bilirubin (µmol/L)	<5.1	12	14	2	61	4	High	NR	2.16	8.0 (2.1 - 25.8)
Indirect bilirubin (µmol/L)	<18	15	14	1	39	3	High	NR	3.24	12.5 ± 14.3
Prothrombin time (s)	10-14	26	15	13	19.7	11.5	NR	NR	12.4	16.3 ± 5.6
INR	0.61-1.17	2.1	1.3	1.1	1.67	0.96	NR	NR	1.1	1.4 ± 0.4
APTT (s)	28-43	38	28	26	27.2	25.1	NR	NR	29	27.6 (25.8 - 31.3)
APTT ratio (s)	0.9-1.4	1.8	0.9	0.71	0.97	0.9	NR	NR	0.93	0.9 (0.8 - 1.4)
AFP (ng/mL)	0-15	3.2	3.1	5.6	26.4	0.8	NR	NR	5.87	4.4 (2.5 - 11.0)
Serum ceruloplasmin (mg/L)	187-322	40	20	0	0.08	Normal	Low	NR	10	14.0 ± 16.7
Serum copper (µg/dL)	11-22	1.1	3.94	2.4	2.3	14.3	Normal	NR	2.7	2.6 (1.9 - 6.5)
Urine copper (UML/24h)	0.24-0.47	30.1	39.66	12.6	15.1	Normal	High	NR	1.07	19.7 ± 15.2

All six patients had high ALT and GGT, while ALP was high in patients 2 and 3. Patient 1 had prolonged prothrombin time (PT), international normalized ratio (INR), and activated partial thromboplastin time ratio. Patient 2 had prolonged PT and high INR at presentation. However, after a few months of diagnosis, he presented with epigastric pain and hematemesis. The repeated coagulation profile also revealed prolonged PT, high INR (1.4), and low platelet count. Patient 4 had high PT and INR. Patients 3, 5, and 8 had normal coagulation profiles. Of the seven patients with available data, five (71.4%) had low serum copper, six (85.7%) had low serum ceruloplasmin, and six (85.7%) had high 24-hour urinary copper.

Regarding genetic testing, the whole exome sequencing test was performed for Patient 1, while the molecular genetic analysis of the ATP7B gene was performed for the other patients. Different ATP7B gene variants in chromosome 13q14.3 were detected in a homozygous state in all patients, while their parents were heterozygous for the same variant. On genetic screening of Family 4 members, three other siblings were heterozygous carriers for the disease. Results of the genetic analysis of patients with WD are shown in Table 3.

**Table 3:** Different ATP7B gene variants on chromosome 13q14.3 of patients with Wilson disease. *missense variants. NR: no record.

Genetic results	Patient 1	Patient 2	Patient 3	Patient 4	Patient 5	Patient 6	Patient 7	Patient 8
Variant	c.3809A>G	c.3061-1G>A	c.3061-1G>A	c.3887A>T	c.2121G>C	c.2121G>C	c.2121G>C	c.2121G>C
Amino acid change^*^	p.Asn1270Ser	NR	NR	p.Asp1296Val	p.Gln707His	p.Gln707His	p.Gln707His	p.Gln707His
Zygosity	Homozygous	Homozygous	Homozygous	Homozygous	Homozygous	Homozygous	Homozygous	Homozygous
Location	Exon 21	Splice site of exon 17	Splice site of exon 17	Exon 21	3’end of exon 7	3’end of exon 7	3’end of exon 7	3’end of exon 7
Classification	Pathogenic	Pathogenic	Pathogenic	Pathogenic	Putative pathogenic	Putative pathogenic	Putative pathogenic	Putative pathogenic

**Figure 2:**
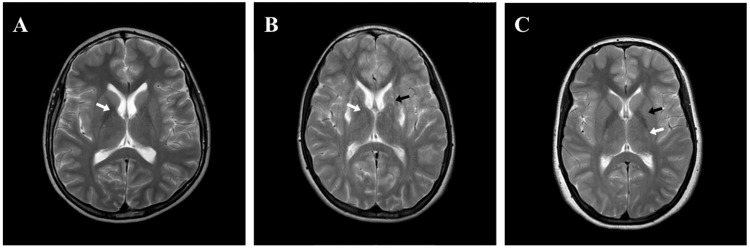
T2-weighted axial brain MRI of patients with Wilson disease. (A) MRI image of Patient 1 showing abnormal hypointense T2 signal intensity at the globus pallidus bilaterally (arrow); (B) MRI image of Patient 2 showing bilateral hyperintensity of the globus pallidus (white arrow) and hypointensity in putamen (black arrow); (C) MRI image of Patient 3 showing high signal intensity at the posterior limb of the internal capsule bilaterally (white arrow) with putamen involvement (black arrow). MRI: magnetic resonance imaging

Regarding radiological imaging, abdominal US revealed coarse hepatic echotexture in five (Patients 1, 2, 4, 5, and 8), signs of liver cirrhosis in four (Patients 1, 2, 6, and 7), mild splenomegaly in three (Patients 2, 3, and 8), while significant ascites, moderate hepatomegaly with fatty infiltration, and mild hepatomegaly were found in patients 1, 5, and 8, respectively. An abdominal CT scan was performed in two patients, which showed mild splenomegaly (Patient 2) and cirrhosis and steatosis (Patient 8). A brain CT scan showed bilateral symmetrical hypodense basal ganglia in Patient 2. Brain MRI was performed in six (75%) patients; three of them (50%) (Patients 1, 2, and 3) had changes in the basal ganglia suggestive of WD (Figure 2).

**Figure 3:**
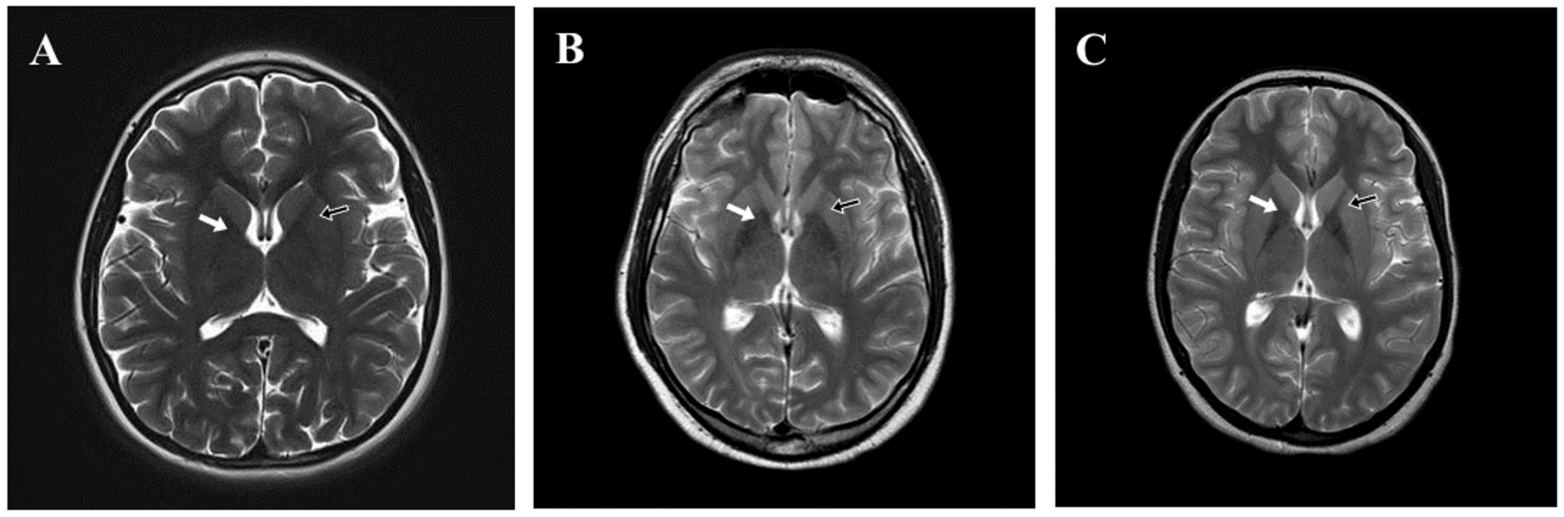
Normal T2-weighted axial brain MRI of patients with Wilson disease. MRI of (A) Patient 4, (B) Patient 5, and (C) Patient 8 showing normal globus pallidus (white arrows) and putamen (black arrows). MRI: magnetic resonance imaging

In addition to the finding shown in Figure 2, Patient 1 had altered increased T2 signal intensity at the posterior limb of the internal capsule, ventrolateral aspect of the thalamus, substantia nigra, and superior colliculi with preservation of the red nucleus. Patient 2 also revealed bilateral basal ganglia hyperdensity along with brainstem lesions suggestive of heavy metal deposition. Nevertheless, three (50%) patients (Patients 4, 5, and 8) had normal brain MRI (Figure 3).

A liver biopsy was performed in two (25%) patients, which revealed hepatic steatosis and hepatic cirrhosis with 90% macrovascular steatosis (Patients 5 and 8, respectively). Upper gastrointestinal endoscopy was performed in three (37.5%) patients (Patients 2, 3, and 8), which revealed Helicobacter pylori macronodular gastritis, mild diffuse gastritis, and mild esophagitis, and nodular gastric mucosa, respectively. One year after the diagnosis, a colonoscopy was performed on one (12.5%) patient (Patient 3) as she developed epigastric pain, hematochezia, and reduced appetite, which revealed a normal colon apart from small hemorrhoids and an anal fissure.

All the patients received dietary modification with a low copper-containing diet and oral chelation therapy (Table 4).

**Table 4:** Medical and surgical therapies of patients with Wilson disease. *died in the hospital while on medical therapy.

Patient management	Patient 1	Patient 2	Patient 3	Patient 4	Patient 5	Patient 6	Patient 7	Patient 8	Total (%)
Dietary restriction	Yes	Yes	Yes	Yes	Yes	Yes	Yes	Yes	8 (100)
D-Penicillamine	Yes	Yes	Yes	Yes	Yes	Yes	Yes	Yes	8 (100)
Pyridoxine (vitamin B6)	Yes	Yes	Yes	Yes	Yes	Yes	Yes	Yes	8 (100)
Vitamin K	Yes	Yes	No	Yes	No	Yes	Yes	Yes	6 (75)
Fresh frozen plasma infusion	No	Yes	No	Yes	No	Yes	Yes	No	4 (50)
Platelets transfusion	No	Yes	No	No	No	Yes	Yes	No	3 (37.5)
Cryoprecipitate	No	No	No	Yes	No	Yes	Yes	No	3 (37.5)
Prednisolone	Yes	Yes	No	No	No	No	No	No	2 (25)
Zinc supplementation	No	Yes	No	No	No	No	No	Yes	2 (25)
Trientine	Yes	No	No	No	No	No	No	No	1 (12.5)
Vitamin B complex	No	No	No	No	No	No	No	Yes	1 (12.5)
Folic acid	No	No	No	Yes	No	No	No	No	1 (12.5)
Liver transplantation	No	Yes	No	No	No	No	No	No	1 (12.5)
Treatment compliance (good)	Good	Poor	Poor	Poor	Good	Good*	Good*	Poor	4 (50)

Patient 1 received steroid therapy initially, which was tapered gradually and then stopped, followed by D-penicillamine 250 mg three times a day and vitamin B6 20 mg once daily. He could not tolerate the D-penicillamine; accordingly, it was changed to trientine. Patient 2 was treated with D-penicillamine 500 mg twice daily and pyridoxine 50 mg once daily. He developed an allergic skin reaction to D-penicillamine, for which prednisolone was given. The Helicobacter pylori infection was treated with triple therapy (amoxicillin, clarithromycin, and omeprazole). Patient 3 and Patient 4 were also kept on D-penicillamine and vitamin B6. Patient 5 was started on D-penicillamine, along with linagliptin, metformin, gliclazide, atorvastatin, Diovan, insulin glargine, insulin aspart, and valsartan. Patient 8 received D-penicillamine, vitamin B complex, and zinc tablets.

On follow-up, six (75%) patients survived (five with medical therapy alone and one with living-related liver transplantation), while two (25%) patients died despite medical therapy (Patients 6 and 7). Patient 1 was stable on trientine alone. One year after the diagnosis, Patient 2 had rapid neurological and hepatic deterioration. Intravenous vitamin K, fresh frozen plasma, and platelet transfusion were required. This deterioration necessitated an overseas liver transplantation (in India), which improved his liver functions but not his neurological condition. He continued to have generalized hypotonia and spasticity (cerebral palsy). Botox injections for upper limb spasticity, baclofen, carbidopa/levodopa, and clonazepam were prescribed. Patient 3 was doing well, but due to poor compliance with D-penicillamine, she started to develop facial and foot numbness, hands shivering with anger, and fine tremors of both hands and arms with action. Due to deranged coagulation, Patient 4 required an infusion of fresh frozen plasma that was stopped immediately after the development of an allergic reaction (urticaria and angioedema). Thereafter, an elevation of hepatic enzymes was noted due to poor compliance with D-penicillamine. Patient 5 developed bilateral facial weakness (Bell’s palsy), but the brain MRI was normal. Patient 6 had a rapidly progressive hepatic encephalopathy, loss of speech, and exaggerated deep tendon reflexes, which subsequently progressed into multisystem involvement and coma. He died while awaiting liver transplantation at the age of 17 years (three years after the diagnosis). Patient 7 lacked a response to the medical treatment, and her case was complicated by hepatic cirrhosis, coagulopathy, hepatic failure, and hepatic encephalopathy. She died at 13 years of age (three years after the diagnosis). Patient 8 was not compliant with her medications, which led to the development of speech difficulty and tremors. At the age of 13 years, she had a road traffic accident that caused fractures of both femurs that required surgical intervention.

## Discussion

This study showed a prevalence of WD in Bahrain of 1.7 patients per 100,000. In comparison, Sandahl et al. (2020) reported an estimated WD prevalence of 1:30,000 to 1:50,000 for the United States, Europe, and Asia [13]. WD is expected to be prevalent in areas with high rates of consanguineous marriages [11], which account for 11.4% in Bahrain [15]. In this study, all the parents were consanguineous (100%). However, Mahmud et al. (2022) [16] and Ali et al. (2021) [17] reported lower parental consanguinity of 38.3% and 64.7% of their patients, respectively. Di Stefano et al. (2012) [4], Bidaki et al. (2012) [18], and Subrahmanyam et al. (2014) [19] also noted parental consanguinity in their case reports. In contrast, Wang et al. (2023) [20], Choudhury et al. (2011) [11], and Zhang et al. (2021) [21] reported unrelated parents.

In this study, the sex distribution was equal (50% each). However, several studies reported a higher incidence of male patients compared to females [17,20,22-24]. Conversely, Eisenbach et al. (2007) reported a greater proportion of females in their research [25]. Nonetheless, many case reports highlighted male patients [4,6,11,18,19,26,27], while others reported female patients [1,2,28]. This variation in sex is typical in autosomal recessive disorders like WD.

WD is most prevalent in patients under 40 years of age but can present at any age [3]. Usually, children and young adults are mostly affected [11]. WD is rarely diagnosed before the age of three [6]. In this study, the mean age at diagnosis was 13 ± 3.6 years (seven patients [87.5%] were diagnosed during adolescence [between 9 and 14 years old], while one patient [12.5%] was diagnosed at 21 years of age). Similarly, Ali et al. (2021) [17] and Day et al. (2021) [24] reported a mean age of 13 ± 4.6 years and a median age of 12.2 years, respectively. However, Zhou et al. (2022) [29], Wang et al. (2023) [20], Aaraj et al. (2021) [22], and Kumar et al. (2022) [23] reported a lower mean age of 5.08 ± 2.06, 7.62 ± 3.46, 9.7, and 10.2 ± 1.8 years, respectively. Other reports represented patients that were diagnosed in infancy (nine months) [6], childhood (7.5 years) [26], adolescence [2,4,7,19], and adulthood [11,18,21,27,28]. Late presentation can be attributed to the time needed for copper accumulation in the body tissues, which can be affected by the variation in dietary consumption of copper-containing foods [1,27].

WD has a variety of clinical presentations [2]. The most common symptoms and signs are hepatic (steatosis, cirrhosis, chronic hepatitis, and acute liver failure), neurologic (ataxia, dysarthria, dystonia, tremors, and Parkinson’s-like symptoms), psychiatric (behavioral, affective, schizophrenia-like type, and cognitive disorders), and ophthalmic (KF rings) [7]. 

Hepatic involvement is the most common presentation in WD [17]. This is in parallel to the finding of our study, where hepatic manifestations accounted for 75% of patients, being the most common presentation, and 50% of patients presented with jaundice. Likewise, several studies reported patients presented with jaundice [11,22,23,29]. In addition, 50% of our patients had hepatomegaly on abdominal examination, while mild splenomegaly was detected in 37.5% of the patients via abdominal US. Several studies reported an isolated hepatomegaly [11,19,23], while others reported an isolated splenomegaly [23,27]. However, Zhang et al. (2018) [28], Aaraj et al. (2021) [22], and Subrahmanyam et al. (2014) [19] reported hepatosplenomegaly. 

In this study, 50% of patients had neurological manifestations, of which ataxic gait was the predominant presentation (37.5%). However, Ali et al. (2021) [17] reported a higher percentage of neurological symptoms in 62.7%. In contrast, Day et al. (2021) [24] reported these manifestations in 36.2% of cases, while Wang et al. (2023) [20] reported only 2.2%. Neurological symptoms can present at the beginning of the disease presentation, along with hepatic involvement, or after years from the disease onset [3]. Furthermore, 37.5% of our patients developed tremors because of poor compliance with medical treatment. Similarly, tremors were reported in other studies [7,17,21,24,27]. Speech difficulty was noted in 25% of our patients in the form of slurred speech or speech loss. Similarly, affection for speech was reported in several previous studies [1,7,23,24,28]. On neurological examination, 25% of our patients had hypotonia, low power, and brisk reflexes. Likewise, Subrahmanyam et al. (2014) [19] reported reduced muscle power in their patient. One of our patients (Patient 3) had a positive Romberg's sign similar to patient-reported by Nikam et al. (2013) [7].

A variety of psychiatric symptoms are associated with WD, including changes in behavior, depression, anxiety, mild cognitive deterioration, or clumsiness [7]. Psychiatric or behavioral manifestations were detected in 50% of our patients, while one patient was initially referred to psychiatric care for being notably introverted, then he started to have neurological manifestations. About half of the patients with WD have shown psychiatric or neurological symptoms [7]. However, Kumar et al. (2022) reported 29.6% of their patients with psychiatric symptoms [23]. Several studies also reported psychiatric symptoms [18,24]. Deterioration of school performance was found in 62.5% of our patients.

Slit lamp examination of the eyes showed bilateral KF rings in 25% of our patients. Most studies reported KF rings in their patients’ eyes [2,7,16-21,23,25]. However, Di Stefano et al. (2012) [4] and Kumar et al. (2010) [27] reported patients without KF rings. The KF rings are due to copper deposits in the Descemet’s layer of the cornea, and a slit lamp is needed for the diagnoses [30]. These rings may disappear with treatment [30]. Diagnosis of WD could be established in the presence of hepatic and neurological symptoms, regardless of the existence of KF rings [27].

Patients’ family screening for the disease is important, as some of their siblings can be homozygous [31]. Some patients with WD were diagnosed during the relative screening [31]. In our study, 37.5% of the patients were diagnosed upon family screening at the ages of 9, 12, and 21 years with no obvious symptoms. Day et al. (2021) [24] and Mahmud et al. (2022) [16] also screened their patients’ families, and WD was detected in the siblings, even though some were asymptomatic.

Regarding laboratory findings in patients with WD, hemolytic anemia was seen in 25% of our patients. Hemolytic anemia may be due to the large amount of copper released after hepatocyte damage, causing acute liver disease [1]. Although Coombs-negative hemolytic anemia is not very common (10% to 15% of cases), it could be the only initial symptom of WD [17]. Nonetheless, Aaraj et al. (2021) reported Coombs-negative hemolytic anemia in 69.2% of their patients [22]. In addition, all our patients (100%) had high ALT and GGT levels, and 33.3% had high ALP levels. Similarly, Di Stefano et al. (2012) [4], Choudhury et al. (2011) [11], Bidaki et al. (2012) [18], and Zhang et al. (2018) [28] reported high ALT levels. Moreover, Bidaki et al. (2012) reported high ALP [18]. At presentation, 50% of our patients had prolonged PT, and 33.3% had high INR. Subrahmanyam et al. (2014) [19], Halimiasl et al. (2013) [26], and Kumar et al. (2010) [27] also reported prolonged PT and high INR in their patients. Likewise, Choudhury et al. reported a high INR [11].

In the present study, diagnosis of WD was confirmed by genetic testing in all patients, along with measuring serum ceruloplasmin, serum copper, and 24-hour urinary copper. Two studies reported low serum ceruloplasmin and high urinary copper in their patients [17,23]. Urinary copper is considered a valuable tool to diagnose WD and monitor treatment efficacy [6]. However, the diagnostic criteria for WD are regularly updated. Three guidelines for diagnosing WD are available: the AASLD guideline issued in 2008 [8], the EASL guideline issued in 2012 [9], and the ESPGHAN guideline issued in 2018 [10]. Initially, AASLD was proposing a clinical/biochemical algorithmic approach, while EASL and ESPGHAN were favoring the use of the Leipzig score. According to the AASLD, the diagnosis depended on a combination of any two of the following three findings as strong evidence for WD: (1) low serum ceruloplasmin concentration; (2) high urinary copper; and (3) increased hepatic copper concentration [32]. EASL guidelines also endorsed the presence of reduced serum ceruloplasmin levels and increased urinary copper excretion. The three guidelines approved the ATP7B gene variant detection as the gold standard for the diagnosis. They also stressed the importance of 24-hour urinary copper measurement and liver copper quantification, either by liver biopsy or noninvasive methods. However, measuring serum ceruloplasmin level alone is no longer considered sufficient for the diagnosis as it can be normal in some patients [8-10]. This was the case with one of our patients, who also had a normal ceruloplasmin level. Moreover, the screening modality for first-degree relatives differs among guidelines. While AASLD and ESPGHAN were recommending clinical and genetic testing, EASL was recommending genetic testing alone. 

More than 1275 different ATP7B gene variants have been discovered to cause WD, including missense, nonsense, frameshift, and splice site variants [5]. These variants can vary among different ethnic groups and populations [5]. While the H1069Q missense variant is the most prevalent in Europe, Northern America, and North Africa, the R778L, C271*, and M645R variants are the most frequently reported in the East Asian, Middle Eastern-South Asian, and South American populations, respectively [5]. In this study, genetic testing was performed for all patients, and the results revealed homozygous variants in the ATP7B gene on chromosome 13q14. Patient 1 had a homozygous missense variant, c.3809A>G (p.Asn1270Ser). According to the Human Gene Mutation Database Professional 2022.1, this variant has previously been described as disease-causing WD by Wu et al., 1999 [33]. ClinVar also listed this variant as pathogenic; variation identification number (ID): 3859 [34]. Patients 2 and 3 were homozygous for the c.3061-1G>A variant. This variant has previously been described as disease-causing for WD by Dastsooz et al., 2013 [35]. ClinVar listed this variant as pathogenic; variation ID: 1452404 [36]. Patient 4 had a homozygous variant for c.3887A>T (p.Asp1296Val). This variant was described earlier in cases with WD by Yamaguchi et al., 2021 [37]. ClinVar listed this variant as pathogenic; variation ID: 161207 [38]. Patients 5, 6, 7, and 8 were homozygous for the putatively pathogenic c.2121G>C (p.Glu707His) variant in exon 7 of the ATP7B gene. This variant has not been previously described in the literature, and there is no information regarding its allele frequency in the general population. According to different bioinformatic tools, it was predicted to be disease-causing.

In this study, radiological imaging revealed liver cirrhosis in 62.5% of patients and ascites in 12.5%. Ali et al. (2021) reported liver cirrhosis and ascites in 9.8% and 11.8% of their patients, respectively [17]. Moreover, Kumar et al. (2022) reported signs of chronic liver disease in 100% of their patients via US imaging [23]. Brain MRI was performed in 75% of our patients, of whom 50% had changes suggestive of WD in the form of bilateral hyperintensities in the globus pallidus, while 50% had normal brain MRI. Radiological abnormalities related to WD have been previously reported in several studies [2,7,19,20,23,27,28]. However, normal brain MRI was also reported by Di Stefano et al. (2012) [4] and Zhou et al. (2022) [29].

Dietary measures via consumption of low-copper foods (i.e., avoidance of intake of chocolate, nuts, liver, and seafood), pharmacotherapy including oral chelators or zinc salts, and for selected patients, liver transplantation are the recommended therapeutic options in patients with WD [1,12]. All patients in this study received dietary restriction advice and oral chelating therapy; 75% of them survived (five with medical therapy alone and one with liver transplantation). Treatment of WD with oral chelating therapy can lead to better survival without the need for liver transplantation [12]. In the present study, despite medical therapy, two patients died due to rapid progression to hepatic encephalopathy. In addition, one of our patients had rapid deterioration of his neurological symptoms, which mandated an overseas liver transplantation. Liver transplantation is considered a rescue factor for patients with WD associated with severe neurological involvement and can improve their survival [12]. Similarly, one of our patients underwent liver transplantation; however, the established neurological impact could not be reversed. Day et al., 2021 also reported liver transplantation in 28% (n=21/74) of their patients, in which 86% of them survived [24]. Nonetheless, all guidelines recommended liver transplantation in patients with acute liver failure due to WD, but not primarily for neuropsychiatric disorders [8-10]. Therefore, the revised King's score should be used to guide transplant listing [9].

This study had several limitations, the main of which was the small number of patients. Nonetheless, this can be explained by the rarity of the disease [2]. Although this is a single-center study, our hospital is the main center in Bahrain and covers the whole country’s population. Moreover, being a retrospective study, the laboratory findings of the two deceased patients could not be fully retrieved, and patients’ adherence to chelation therapy could not be quantified. Even with these limitations, the findings of this study are important, being the first WD report from Bahrain. These findings highlight the importance of early investigation of patients with psychiatric and neurological symptoms, along with elevated liver enzymes, before they progress to irreversible hepatic and neurological morbidities or even mortality [17].

## Conclusions

WD is a rare disorder in Bahrain. This study documents eight patients with WD from four families; five of them were symptomatic at presentation, while three were diagnosed through genetic family screening. Hepatic involvement was the most common presentation, followed by behavioral changes and neurological symptoms. Two patients had KF rings, while three had classical brain MRI findings. Genetic testing served as a confirmatory diagnostic tool that revealed a diversity of variants causing the disease in Bahrain, of which one variant was a novel mutation. Despite dietary restrictions and oral chelating therapy, one patient required liver transplantation, and two died, indicating the high morbidity and mortality associated with this treatable disease. The small sample size is the main limitation of this study, reflecting the rarity of the disease in our country. Further prospective multicenter studies with a larger study population are needed, focusing on phenotype-genotype correlation in patients with WD, patients’ compliance with oral chelating therapy, and the long-term outcomes of those who survived. 
